# Critical appraisal of a pilot study examining a ketogenic diet as an adjunct therapy in college students with major depressive disorder

**DOI:** 10.1038/s41398-026-03809-w

**Published:** 2026-01-21

**Authors:** Muhammad Khizar, Muhammad Zaib, Hasiba Karimi, Hasibullah Aminpoor

**Affiliations:** 1 Faculty of Medicine, Georgian American University, Manifest Medical Research Co, Tbilisi, Georgia; 2 Faculty of Medicine, Bezmialem Vakif University, Istanbul, Turkey; 3 Faculty of Medicine, Kabul University of Medical Sciences “Abu Ali Ibn Sina”, Kabul, Afghanistan

**Keywords:** Depression, Physiology

## Abstract

Major depressive disorder (MDD) remains a leading cause of disability worldwide, and innovative adjunctive strategies are needed to enhance treatment outcomes. This critical appraisal examines a recent pilot study by Decker et al., which evaluated a 10–12 week well-formulated ketogenic diet (WFKD) as an adjunct therapy for college students with MDD. In this uncontrolled cohort (n = 16 completers), mean PHQ-9 and HRSD scores decreased by approximately 69–71% (p < 0.001), accompanied by notable improvements in self-reported wellbeing, cognitive performance, body composition, and metabolic biomarkers (e.g., leptin reduction, BDNF increase). These findings suggest that metabolic interventions may exert clinically meaningful antidepressant effects comparable to conventional therapies. However, as a single-arm study with a small, self-selected sample, causality cannot be established, and placebo effects or concurrent counseling may have contributed to outcomes. The authors appropriately call for larger, randomized controlled trials with longer follow-up and diverse populations to confirm efficacy, explore underlying mechanisms (e.g., neuroinflammation, gut–brain axis modulation), and optimize implementation. If validated, integrating dietary strategies into psychiatric and college counseling programs could offer a low-risk, holistic approach to improving mental health outcomes.

**To the Editor**:

We read with great interest the recent pilot study by Decker et al. [1], who report that a 10 - 12 week well-formulated ketogenic diet (WFKD) was feasible and associated with marked symptom improvements in college students with major depressive disorder (MDD). In this uncontrolled cohort (n = 16 completers), mean PHQ-9 and HRSD scores fell **~69–71%** by study end (p < 0.001) [1]. These dramatic reductions along with nearly threefold gains in self-reported wellbeing and improvements in cognitive task performance suggest that metabolic interventions merit serious attention as adjuncts in depression treatment [1]. Notably, the WFKD also yielded metabolic benefits (6.2% body mass loss, : 13% fat loss) and hormonal changes (52% leptin, + 32% BDNF) [1], supporting a growing view that disturbed energy homeostasis contributes to mood disorders.

Decker et al.’s findings align with emerging evidence that diet can influence mood. Although high-quality trials are sparse, several uncontrolled studies have hinted at antidepressant effects of low-carbohydrate or ketogenic regimens [2]. For example, a recent systematic review noted possible benefits of ketogenic interventions in bipolar disorder and unipolar depression though it emphasized the need for robust trials [2]. More broadly, meta-analyses show that general dietary improvement can modestly reduce depressive symptoms [3]. In this context, the pilot results [1] are noteworthy in quantifying the potential magnitude of change (roughly equivalent to effects seen with standard therapies) and by documenting feasibility in a real-world college setting. The work thus contributes valuable preliminary data to the field of nutritional psychiatry.

We also note important limitations. As a single-arm study, it cannot disentangle the effects of the diet from concurrent counseling, placebo effects or natural recovery [1]. The small, self-selecting sample (students motivated to try a diet intervention) may overestimate efficacy. Decker et al. appropriately call for randomized controlled trials [1]; given reports that symptoms can return after diet discontinuation [2], longer follow-up is also crucial. Future work should assess diverse populations, control diets (e.g. healthy low-fat comparison), and objective adherence metrics.

In spite of these caveats, the study has broad implications. It reinforces the link between metabolic health and mood depression and metabolic syndrome are known to be bidirectionally related [4] and suggests that addressing metabolism could “kill two birds” by improving both mental and physical health. If validated, such dietary strategies could be integrated into college counseling programs or general psychiatric care, augmenting traditional therapies with low-risk lifestyle modification. This interdisciplinary approach involving psychiatrists, dietitians, and public health experts could help tailor preventive and therapeutic guidelines that promote mental wellness through nutrition.

It is also important to note that many participants were receiving standard therapies, including counseling and pharmacological treatment. While the pilot study suggested no marked differences between medicated and unmedicated individuals, the small sample size limits firm conclusions. Future research should continue to assess the ketogenic diet as an adjunctive intervention, carefully evaluating its effects alongside established treatments

In conclusion, Decker et al. [1] provide an impressive proof of concept that a ketogenic diet may alleviate depressive symptoms in young adults, even when used as an adjunct to therapy and medications. Their results should encourage further collaboration and rigorous research. Larger, controlled trials are urgently needed to confirm efficacy, examine mechanisms (e.g., neuroinflammation, gut-brain axis, BDNF modulation), and optimize implementation. Continued exploration of nutritional therapies holds promise for enhancing mental health outcomes and informing policy on holistic care strategies.

## References

[1] Decker DD, Patel R, Cheavens J, Hayes SM, Whitted W, Lee AJ, et al. A pilot study examining a ketogenic diet as an adjunct therapy in college students with major depressive disorder. Transl. Psychiatry. 2025;15:322. 10.1038/s41398-025-03544-8. 40925905 10.1038/s41398-025-03544-8 PMC12420795

[2] Dietch DM, Kerr-Gaffney J, Hockey M, Marx W, Ruusunen A, Young AH, et al. Efficacy of low carbohydrate and ketogenic diets in treating mood and anxiety disorders: systematic review and implications for clinical practice. BJPsych Open. 2023;9:e70. 10.1192/bjo.2023.36. 37066662 10.1192/bjo.2023.36 PMC10134254

[3] Firth J, Marx W, Dash S, Carney R, Teasdale SB, Solmi M, et al. The effects of dietary improvement on symptoms of depression and anxiety: a meta-analysis of randomized controlled trials. Psychosom. Med. 2019;81:265–80. 10.1097/PSY.0000000000000673. 30720698 10.1097/PSY.0000000000000673 PMC6455094

[4] Pan A, Keum N, Okereke OI, Sun Q, Kivimaki M, Rubin RR, et al. Bidirectional association between depression and metabolic syndrome: a systematic review and meta-analysis of epidemiological studies. Diabetes Care. 2012;35:1171–80. 10.2337/dc11-2055. 22517938 10.2337/dc11-2055 PMC3329841

